# Optimisation of Animal Handing and Timing of 2-deoxy-2-[^18^F]fluoro-D-glucose PET Tumour Imaging in Mice

**DOI:** 10.1007/s11307-024-01956-4

**Published:** 2024-11-11

**Authors:** Richard L. Hesketh, David Y. Lewis, Kevin M. Brindle

**Affiliations:** 1 Cancer Research UK Cambridge Institute, Robinson Way, Cambridge, CB2 0RE UK; 2 Centre for Medical Imaging, University College London, Charles Bell House, 43-45 Foley Street, London, W1W 7TY UK; 3 Cancer Research UK Scotland Institute, Garscube Estate, Switchback Road, Glasgow, G61 1BD UK; 4 School of Cancer Sciences, University of Glasgow, Glasgow, G61 1QH UK

**Keywords:** PET, [^18^F]FDG, Timing, Standardisation, Optimisation, Dynamic

## Abstract

**Purpose:**

In humans, 2-deoxy-2-[^18^F]fluoro-D-glucose ([^18^F]FDG) tumour-to-background contrast continues to increase long after a typical uptake period of 45 – 60 min. Similar studies have not been performed in mice and the static imaging time point for most studies is arbitrarily set at 30 – 60 min post-injection of [^18^F]FDG. Ideally, static PET imaging should be performed after the initial period of rapid uptake but this period has not been defined in mice, with previous dynamic studies in mice being limited to 60 min. This study aimed to define the kinetics of [^18^F]FDG biodistribution over periods of 3 – 4 h in different murine tumour models, both subcutaneous and autochthonous, and to further refine fasting and warming protocols used prior to imaging.

**Procedures:**

Dynamic [^18^F]FDG PET-CT scans lasting 3 or 4 h were performed with C57BL/6 J and Balb/c nude mice bearing subcutaneous EL4 murine T-cell lymphoma and Colo205 human colorectal tumours, respectively, and with transgenic Eμ-*Myc* lymphoma mice. Prior to [^18^F]FDG injection, four combinations of different animal handling conditions were used: warming for 1 h at 31 °C; maintenance at room temperature (20 – 24 °C), fasting for 6 – 10 h and a fed state.

**Results:**

Tumour mean standardised uptake value (SUV_mean_) peaked at 147 ± 48 min post injection in subcutaneous tumours and 74 ± 31 min in autochthonous Eμ-*Myc* lymphomas. The tumour-to-blood ratio (TBR) peaked at 171 ± 57 and 83 ± 33 min in subcutaneous and autochthonous Eμ-*Myc* tumours, respectively. Fasting increased tumour [^18^F]FDG uptake and suppressed myocardial uptake in EL4 tumour-bearing mice. There was a good correlation between tumour SUV_mean_ and *K*_*i*_ calculated using an input function (IDIF) derived from the inferior vena cava.

**Conclusions:**

Delayed static [^18^F]FDG-PET imaging (> 60 min) in both autochthonous and subcutaneous tumours in improved tumour-to-background contrast and increased reproducibility.

**Supplementary Information:**

The online version contains supplementary material available at 10.1007/s11307-024-01956-4.

## Introduction

Positron emission tomography (PET) with 2-deoxy-2[^18^F]fluoro-D-glucose ([^18^F]FDG) is widely used as a surrogate measure of glucose uptake into tissues which can be increased in a range of pathologies including inflammation and malignancy. The ubiquitous clinical use of [^18^F]FDG-PET, particularly in oncology, has made it the gold standard for comparing the performance of new functional contrast agents. However, the uptake of [^18^F]FDG is affected by numerous factors attributable to variations in biology, scanner and hardware performance, acquisition and reconstruction settings and methods of analysis. Inadequate control of these variables can result in poor repeatability and reproducibility, limiting study comparability and potentially leading to erroneous conclusions.

Clinical guidelines such as those produced by the European Association of Nuclear Medicine (EANM) strived to standardise many of these factors [1]. For example, fasting for at least 4 h prior to injection, maintaining a warm environment (about 24 °C) for 30 – 60 min prior to injection, minimising physical activity during the uptake period, and a static PET acquisition starting between 45 – 60 min post injection (p.i.) are commonly employed. Several clinical studies have shown that the tumour-to-blood ratio (TBR), and consequently tumour-to-background contrast, continues to increase over time, up to 8 h in glioma patients and 5 h in lung cancer patients [2, 3]. The recent introduction of total-body PET into clinical practice makes delayed and dynamic imaging more practical and therefore the debate regarding optimum time to perform imaging is being reconsidered [4].

In contrast, protocols for pre-clinical small animal imaging lack standardisation, with a multitude of potential biological and technical variables. Early investigations recommended warming before [^18^F]FDG injection and throughout the uptake period and fasting and anaesthesia (preferably using isoflurane) during the uptake and scanning periods, practices which have been widely adopted [5]. The optimal timing of static PET acquisitions in mice has received limited attention, with static imaging typically commencing between 30—60 min post injection, as in clinical studies.

We hypothesised that static imaging at later time points would improve tumour-to-background contrast and potentially improve repeatability by imaging in the plateau phase rather than the initial rapid uptake phase. Additionally, we postulated that the rate of uptake would vary between autochthonous and subcutaneous tumours. To investigate this, dynamic [^18^F]FDG-PET scans (3—4 h) were performed in two subcutaneous xenograft models (EL4 mouse T cell lymphoma and Colo205 human colorectal cancer) and an autochthonous lymphoma model (Eμ-*Myc*). In EL4 tumour-bearing mice, we investigated the effects of fasting and warming animals prior to [^18^F]FDG injection on tracer kinetics.

## Methods

Animal experiments were performed in compliance with a project licence issued under the Animals (Scientific Procedures) Act of 1986. Protocols were approved by the Cancer Research UK, Cambridge Institute Animal Welfare and Ethical Review Body.

### Cell Culture and Tumour Growth

EL4 TIB-39 murine T-cell lymphoma cells and Colo205 metastatic colon adenocarcinoma cells (ATCC, VA, USA) were cultured in RPMI 1640 medium (Gibco, MA, USA) supplemented with 10% FBS and 2 mM glutamine. Cells (5 × 10^6^) were resuspended into 0.2 mL PBS and implanted into the flanks of mice (Charles River, MA, USA); EL4 cells into adult female C57BL/6 J mice (10 – 32 weeks, 25 ± 5 g) and Colo205 cells into BALB/c nude mice (10 – 24 weeks; 21 ± 3 g). Tumours were imaged when they were 0.8 – 1.7 cm^3^.

Heterozygous Eμ-*Myc* transgenic mice were bred and the presence of the *c-Myc* transgene and *IgH* enhancer confirmed by real-time polymerase chain reaction for the transgene sequence CCAGCCTCAATCTCA. The forward and reverse primer sequences were CCAGATATTGAAGCAGAACGCAAAA and CAAAACGTCGGCTACAGTAACTTT, respectively (Transnetyx, TN, USA). Adult mice were assessed twice weekly for spontaneous development of cervical and axillary lymphadenopathy and imaged mice were aged 8 – 24 weeks and weighed 29 ± 5 g.

### Animal Handling

Animal handling conditions were varied in EL4 tumour-bearing mice according to Table 1, with conditioning beginning in the morning (between 05:00 and 08:00) [6]. Water was freely available at all times for all mice. Fasted mice were transferred to a new cage without food for 6 – 10 h [7]. Mice in the fed group were transferred to a new cage 6 – 10 h prior to imaging with access to food *ab libitum*. For warming mice were placed in a chamber heated to 31 °C, the thermoneutral temperature of mice, for 60 min prior to anaesthesia. Mice not warmed remained at 18 – 21 °C, the ambient temperature of the animal house, up to induction of anaesthesia. Colo205 tumour-bearing mice (*n* = 8) were imaged following a period of fasting and warming. Eμ-*Myc* mice (*n* = 4) were warmed but were fed.

**Table 1:** Tumour types and animal handling conditions prior to injection

Tumour Type	Number of mice	Conditions prior to [^18^F]FDG injection	Blood glucose (mmol/L)
EL4	3	Fasting, warmed 31 ºC	5.83 ± 0.95
EL4	5	Fasting, room temperature (RT)	4.87 ± 1.61
EL4	5	Fed, warmed 31 ºC	7.28 ± 1.43
EL4	3	Fed, RT	9.37 ± 1.50
Colo205 fasted, warmed	8	Fasting, warmed 31 ºC	8.39 ± 1.84
Eµ-*Myc* fed, RT	4	Fed, warmed 31 ºC	9.20 ± 1.85

Mice were anaesthetised by inhalation of 1 – 2.5% isoflurane (Isoflo, Abbotts Laboratories, UK) in a 50:50 mix of air (1 L/min) and oxygen (1 L/min). Following induction of anaesthesia rectal temperature was maintained between 36 and 37.5 °C using warmed air flow and anaesthesia was adjusted to maintain a respiratory rate of 80 – 120 breaths per minute. Venous glucose concentration was measured using a StatStrip Xpress glucose meter (Nova Biomedical, MA, USA) prior to imaging.

### [^18^F]FDG-PET Imaging

Dynamic 3 h (Colo205 and Eμ-*Myc*) and 4 h (EL4) PET scans were acquired using a Nanoscan PET/CT (Mediso, Hungary) and were started immediately prior to intravenous injection over 30 s of 12.8 ± 4.2 MBq [^18^F]FDG. Data were acquired in list-mode format and reconstructed with an isotropic voxel size of 0.6 mm using a 3D ordered-subset expectation maximisation method, two iterations and six subsets. Images were normalised and corrected for decay, dead-time, random events and attenuation. Computed tomography (CT) images were acquired for anatomic co-registration and attenuation correction. Scans, 3 and 4 h, were reconstructed into 91 and 97 time frames, respectively (5 s × 24, 0 – 2 min; 15 s × 12, 2 – 5 min; 30 s × 10, 5 – 10 min; 1 min × 10, 10 – 20 min; 2 min × 20, 20 – 60 min; 5 min × 6, 60 – 90 min and 10 min × 9 or 15, 90 min onwards). For Patlak analysis the data were reconstructed in 2 min time frames throughout.

The images were analysed using Vivoquant 3.0 software (InviCRO, MA, USA). An image derived arterial input function (AIF) was acquired from a 3D ROI drawn manually over the aorta / inferior vena cava. Tumour, myocardial and Harderian ROIs were defined on the last timepoint using Otsu and 75%_max_ thresholding, respectively. Liver and kidney ROIs were manually drawn on PET images at 1 min post-injection. Brain ROIs were drawn on the CT. SUV was calculated as:

$$\text{SUV } = \frac{{\text{c}}_{\text{img}}}{\text{ID/BW}}$$

where c_img_ is the activity concentration (MBq / mL) derived from the image ROI, ID is the injected dose (MBq) and BW is the body weight (g) of the animal.

Patlak graphical analysis was used to estimate the net rate of [^18^F]FDG uptake (*K*_*i*_) from the linear portion of the graph between 20 and 60 min [8]. Patlak multiple-time graphical analysis assumes irreversible uptake of a tracer into a tissue compartment. The rate of accumulation of tracer in a tissue ROI (R) at a time (t) after tracer injection can be expressed as:

$$R\left(t\right)={K}_{i}{\int }_{0}^{t}{C}_{p}(\tau )d\tau +{V}_{0}{C}_{p}(t)$$

where ${\text{C}}_{\text{p}}$ is the plasma activity, V_0_ is the blood and interstitial volume (the reversible compartment) of the ROI. This can be rearranged as:

$$\frac{R(t)}{{C}_{p}(t)}={K}_{i}\frac{{\int }_{0}^{t}{C}_{p}(\tau )d\tau }{{\text{C}}_{\text{p}}\left({\text{t}}\right)}+{V}_{0}$$

When plotted graphically, the slope becomes linear after the free [^18^F]FDG in the reversible compartments has equilibrated with the [^18^F]FDG in the plasma and has a gradient equal to *K*_*i*_.

### Statistical Analysis

Graphical and statistical analyses were performed using Prism v6.0 (Graphpad Software, La Jolla, CA, USA). Statistical tests performed were paired or unpaired two-tailed *t*-tests with errors representing standard deviation, unless stated otherwise. *P* values are summarised in figures as: < 0.0001, ****; 0.0001 – 0.001, ***; 0.001 – 0.01, **; 0.01 to 0.05, *.

## Results

**Fig. 1:**
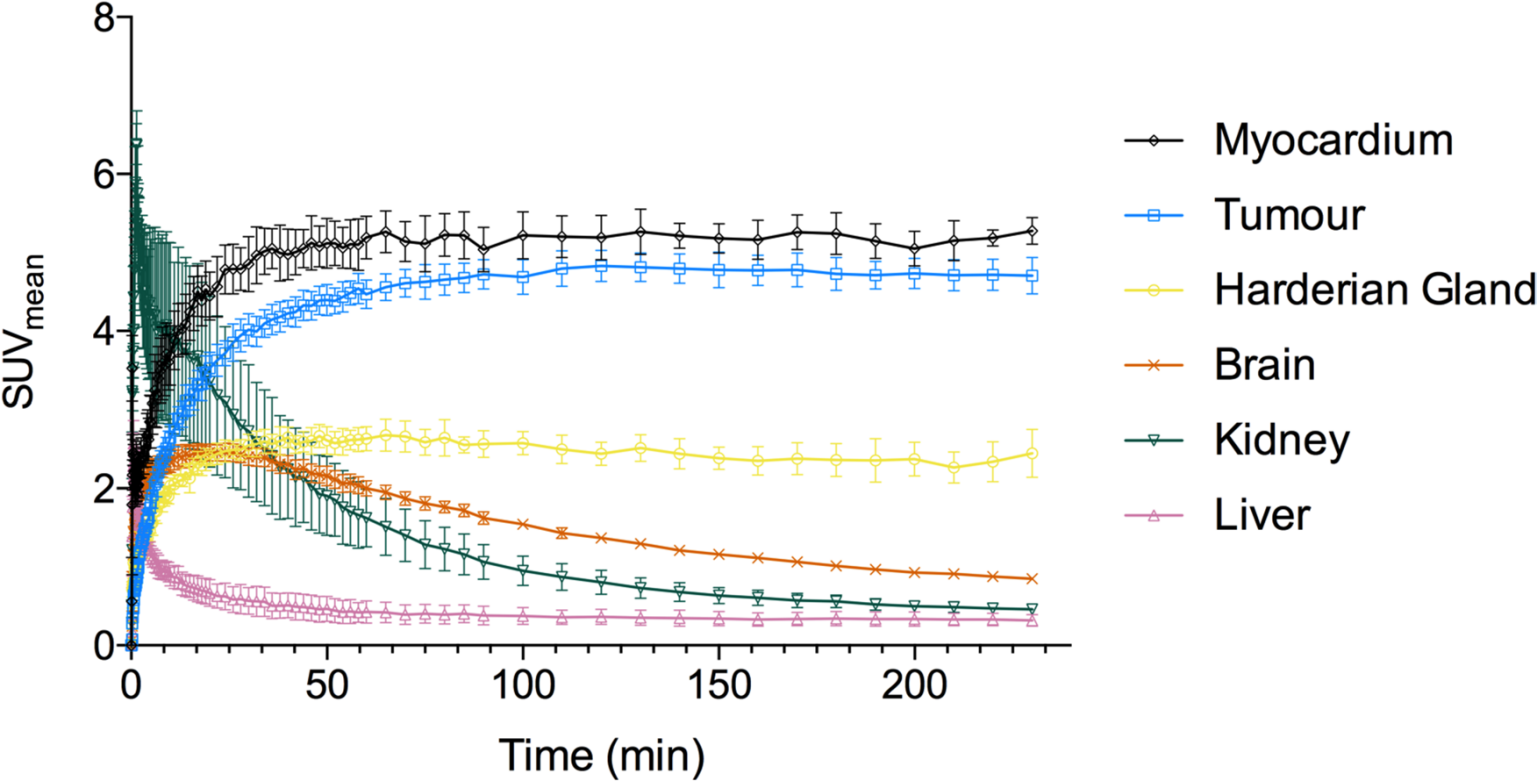
Dynamic [^18^F]FDG-PET measurements acquired over 240 min in warmed and fasted EL4 tumour-bearing mice time activity curves (SUV_mean_) in different organs

Twenty eight mice were injected with 12.75 ± 4.2 MBq [^18^F]FDG (Table 1). Fasting decreased blood glucose concentration in EL4 tumour-bearing mice, from 8.3 ± 1.8 mmol/L (*n* = 8) to 5.2 ± 1.4 mmol/L (*P* = 0.0029, *n* = 8; Table 1). Warming prior to [^18^F]FDG injection had no significant effect on blood glucose concentration. The majority of irreversible [^18^F]FDG uptake was observed in tumours, myocardium and Harderian glands. Significant brown fat and muscle uptake was not reproducibly observed in any cohort. The liver and brain [^18^F]FDG signals were dominated by perfusion but showed a small quantity of irreversible uptake with influx rate constants (*K*_*i*_) of 0.001 min^−1^ and 0.011 min^−1^, respectively (Fig. 1, 2 & S1).

**Fig. 2:**
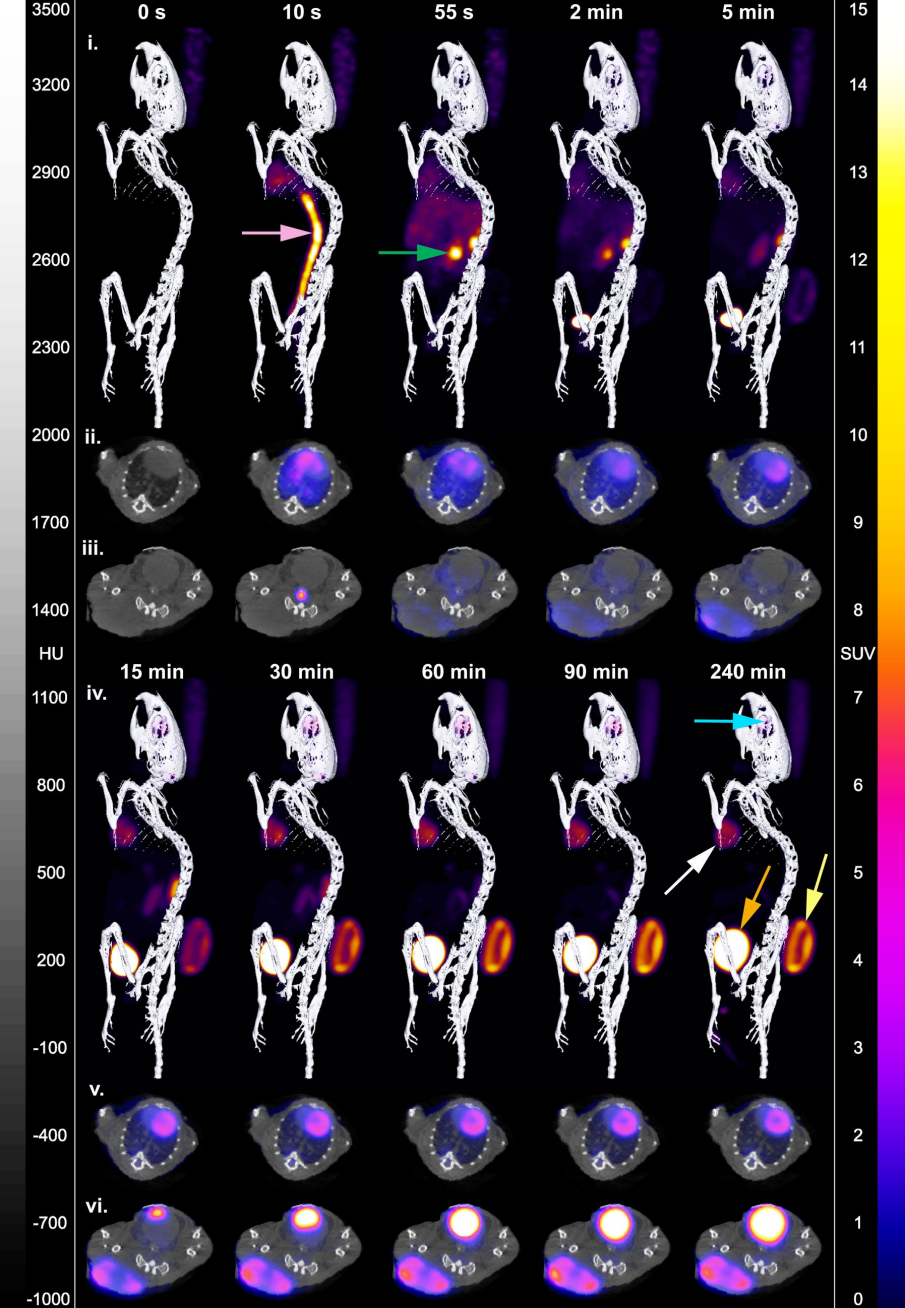
Example dynamic [18F]FDG-PET images of a warmed and fasted EL4 tumour-bearing mouse. (i, iv) Whole body 3D reconstructions and (ii, iii, v, vi) axial slices of [^18^F]FDG-PET CT images through the heart and tumour. Arrows: 10 s, pink arrow – inferior vena cava / aorta; 55 s, green arrow – right kidney; 240 s, blue arrow – Harderian gland; white arrow – myocardium; orange arrow – bladder; yellow arrow – tumour

### Fasting Supressed Myocardial Uptake and Increased Tumour Uptake

**Fig. 3:**
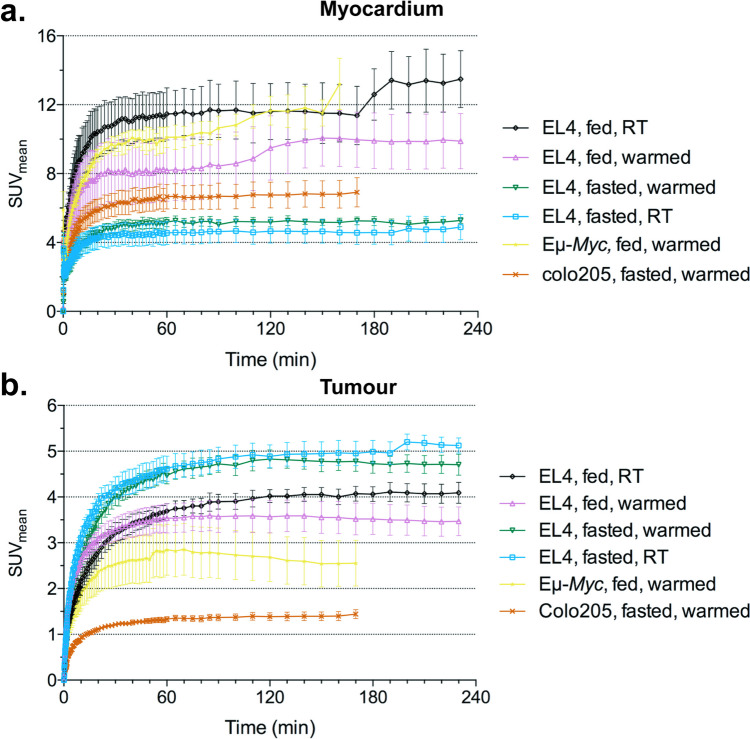
Comparison of [^18^F]FDG uptake under different fasting and warming conditions (**a**) myocardial and (**b**) tumour uptake. The increase in myocardial uptake in EL4 fed, RT mice at approximately 180 min was the result of movement. RT; room temperature

Fasting suppressed myocardial [^18^F]FDG uptake and increased tumour uptake (Fig. 3a). In warmed EL4 tumour-bearing mice fasting decreased myocardial SUV_mean_ from 9.88 ± 3.59 to 5.28 ± 0.29 (*P* = 0.07) while tumour SUV_mean_ increased from 3.47 ± 0.70 to 4.71 ± 0.40 (*P* = 0.03). In EL4 tumour-bearing mice maintained at RT fasting decreased myocardial SUV_mean_ from 13.5 ± 2.86 to 4.9 ± 1.65 (*P* = 0.0015) and increased tumour SUV_mean_ from 4.09 ± 0.40 to 5.13 ± 0.37 (*P* = 0.01). The inverse relationship between cardiac and tumour [^18^F]FDG uptake caused by fasting (-87.2% vs. 26.3%) and unchanged blood [^18^F]FDG activity highlights the heart’s ability to utilise other metabolic substrates, particularly fatty acids.

Warming mice prior to [^18^F]FDG injection did not have a significant effect on tumour or cardiac uptake (Table 2). However, warming and fasting did reduce inter-mouse variability, as indicated by the coefficient of variance (CV) for the SUV_mean_ measurements (Table 2). Eμ-*Myc* tumours had the largest inter-mouse variability with a CV of 0.39. Only Colo205 tumours demonstrated a progressive increase in variability with increasing time post-injection, with the CV increasing from 0.10 to 0.20 from 30 to 170 min p.i. (Fig. S2).

**Table 2:** Tumour SUV_mean_, coefficient of variance of SUV_mean_ and tumour-to-blood ratios (TBR) over time. TBR, Tumour to-blood SUV_mean_ ratio. TTP, time to peak. Error is reported as SD and significant increases from the previous time point are indicated using a ratio paired t-test. *The myocardial data from one Eµ-*Myc* mouse was excluded due to spillover from an adjacent mediastinal mass

Tumour / Conditions	Metric	30 min	60 min	90 min	170 min	230 min	TTP (min)	Mean CV
EL4 fasted, warmed	Tumour SUV_mean_	4.0 ± 0.5	4.5 ± 0.2^*^	4.7 ± 0.2^**^	4.8 ± 0.2^ ns^	4.7 ± 0.4^ ns^	130 ± 20	
	CV (SUV_mean_)	0.087	0.070	0.067	0.079	0.084		0.077
	TBR	5.8 ± 2.9	9.6 ± 5.7^*^	12.5 ± 7.7^**^	16.6 ± 8.6^*^	18.4 ± 9.9^*^	216.7 ± 23.1	
	CV (TBR)	0.49	0.59	0.62	0.52	0.54		0.55
	Myocardial SUV_mean_	4.9 ± 0.6	5.2 ± 0.5^ ns^	5.1 ± 0.5^ ns^	5.3 ± 0.4^ ns^	5.3 ± 0.3^ ns^	125 ± 57.7	
EL4 fasted, RT	Tumour SUV_mean_	4.2 ± 0.5	4.6 ± 0.6^***^	4.8 ± 0.6^**^	5.0 ± 0.6^*^	5.1 ± 0.4^ ns^	190 ± 28.3	
	CV (SUV_mean_)	0.13	0.13	0.12	0.12	0.073		0.11
	TBR	10.3 ± 4.4	13.9 ± 6.2^**^	16.38 ± 7.1^**^	18.9 ± 6.8^*^	19.1 ± 7.6^ ns^	206 ± 37.8	
	CV (TBR)	0.42	0.45	0.43	0.36	0.40		0.41
	Myocardial SUV_mean_	4.4 ± 1.4	4.6 ± 1.5^ ns^	4.6 ± 1.5^ ns^	4.6 ± 1.5^ ns^	4.9 ± 1.7^ ns^	134 ± 51.8	
EL4 fed, warmed	Tumour SUV_mean_	3.3 ± 0.6	3.5 ± 0.7 ^**^	3.6 ± 0.8^ ns^	3.5 ± 0.8^ ns^	3.5 ± 0.7^ ns^	119 ± 32.5	
	CV (SUV_mean_)	0.17	0.20	0.22	0.22	0.20		0.20
	TBR	5.7 ± 3.0	7.5 ± 5.7^ ns^	8.2 ± 6.8^ ns^	7.7 ± 7.5^ ns^	8.1 ± 7.9^*^	108.4 ± 90.2	
	CV (TBR)	0.52	0.76	0.82	0.97	0.97		0.81
	Myocardial SUV_mean_	8.1 ± 3.9	8.2 ± 4.0^ ns^	8.4 ± 4.1^ ns^	10.0 ± 3.6^ ns^	9.9 ± 3.6^ ns^	131.2 ± 62.1	
EL4 fed, RT	Tumour SUV_mean_	3.2 ± 0.4	3.7 ± 0.3^*^	3.9 ± 0.3^*^	4.1 ± 0.3^ ns^	4.1 ± 0.4^ ns^	190 ± 40	
	CV (SUV_mean_)	0.12	0.077	0.072	0.068	0.097		0.087
	TBR	4.7 ± 2.2	7.5 ± 4.7^*^	8.4 ± 5.1^ ns^	10.9 ± 6.8^ ns^	10.6 ± 6.3^ ns^	190 ± 26.5	
	CV (TBR)	0.47	0.62	0.61	0.62	0.59		0.58
	Myocardial SUV_mean_	10.9 ± 2.4	11.5 ± 2.6^ ns^	11.7 ± 2.7^**^	11.4 ± 2.9^ ns^	13.5 ± 2.9^ ns^	168.3 ± 74.9	
Colo205	Tumour SUV_mean_	1.2 ± 0.1	1.3 ± 0.2^****^	1.4 ± 0.2^ ns^	1.4 ± 0.3^ ns^		127.5 ± 51.4	
	CV (SUV_mean_)	0.11	0.12	0.15	0.20			0.14
	TBR	2.5 ± 0.3	4.1 ± 0.8^****^	4.6 ± 0.9^***^	5.6 ± 1.3^***^		165 ± 10.7	
	CV (TBR)	0.13	0.19	0.18	0.23			0.18
	Myocardial SUV_mean_	6.2 ± 1.8	6.7 ± 1.9^***^	6.7 ± 2.0^ ns^	6.9 ± 2.3^ ns^		128 ± 47.0	
Eµ-*Myc*	Tumour SUV_mean_	2.5 ± 0.5	2.8 ± 0.6^*^	2.8 ± 0.6^ ns^	2.6 ± 0.5^ ns^		74 ± 30.7	
	CV (SUV_mean_)	0.39	0.39	0.40	0.39			0.39
	TBR	3.7 ± 2.1	4.8 ± 3.2^*^	4.4 ± 2.6^ ns^	3.4 ± 1.9^ ns^		83.8 ± 33.0	
	CV (TBR)	0.6	0.7	0.6	0.5			0.59
	Myocardial SUV_mean_*	9.7 ± 0.8	9.9 ± 1.3^ ns^	10.3 ± 1.2^ ns^	13.2 ± 2.6^ ns^		170 ± 0	

### Temporal Changes in Tumoural [^18^F]FDG Uptake

**Fig. 4:**
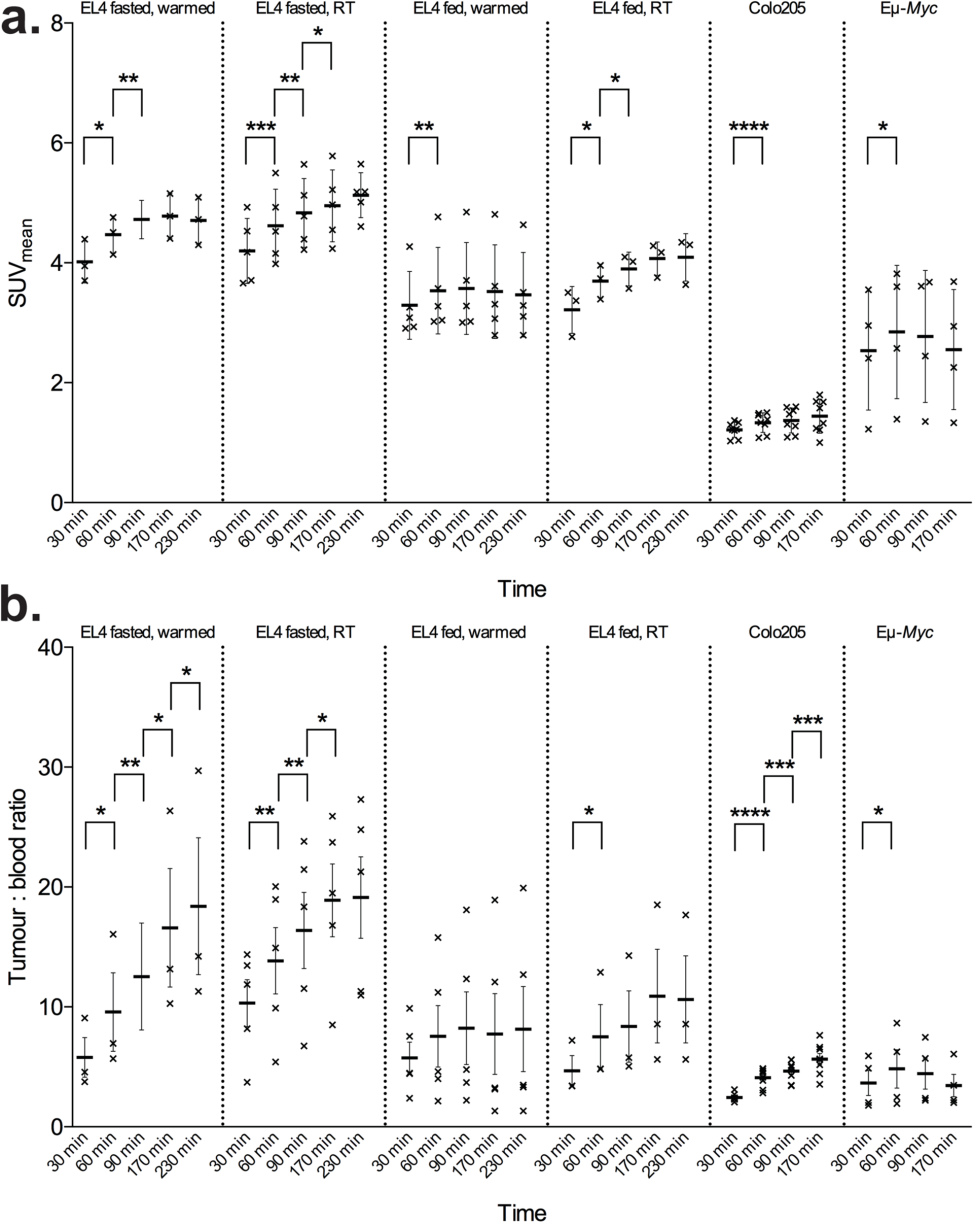
Comparison of tumour SUV_mean_ and tumour-to-blood ratios at different times post-injection of [^18^F]FDG in different mouse models and handling conditions. Error bars represent SEM

SUV_mean_ peaked at 147 ± 48 min in all subcutaneous tumours and 74 ± 31 min in Eµ-*Myc* cervical tumours (Table 2). All mice demonstrated a rapid increase in tumour [^18^F]FDG concentration between 0 – 30 and 30 – 60 min (Table 2, Fig. 4a). Between 30 – 60 min SUV_mean_ increased by 10.4 ± 4.5% across all tumour types (Table 3). Thereafter, the rate of change decreased. In EL4 tumours under all handling conditions the percentage change in SUV_mean_ was 3.9 ± 2.6% between 60 – 90 min and 1.3 ± 3.2% between 90 – 170 min. In colo205 tumours the percentage change in SUV_mean_ was 2.6 ± 4.0 and 4.5 ± 8.2% between 60 – 90 and 90 – 170 min, respectively. In Eµ-*Myc* mice cervical tumour [^18^F]FDG uptake increased by 12.4 ± 7.0% between 30 – 60 min, and then decreased by -2.8 ± 3.5% and -7.0 ± 8.4% between 60 – 90 min and 90 – 170 min, respectively. Three of the four Eµ-*Myc* tumours plateaued after 60 min, whereas in one mouse SUV_mean_ decreased from a peak of 3.8 at 56 min to 2.9 at 170 min. This decrease could not be explained by an inaccurate ROI placement.

**Table 3:** Percentage change of SUV_mean_ between scan times

Tumour / conditions	30—60 min	60—90 min	90—170 min	170—230 min
Colo205	9.6 ± 3.1	2.6 ± 4.0	4.5 ± 8.2	
Eµ-*Myc*	12.4 ± 7.0	-2.7 ± 3.5	-7.0 ± 8.4	
EL4 fasted, warmed	11.4 ± 3.0	5.7 ± 0.9	1.2 ± 1.1	-1.6 ± 0.7
EL4 fed, warmed	7.0 ± 3.5	0.8 ± 2.0	-1.5 ± 3.8	-1.2 ± 2.0
EL4 fasted, RT	9.9 ± 2.6	4.8 ± 1.4	2.5 ± 1.4	4.3 ± 10.2
EL4 fed, RT	15.4 ± 6.2	5.5 ± 2.0	4.5 ± 2.4	0.4 ± 3.2

TBR peaked at 171.3 ± 57 min in all subcutaneous tumours across all conditions. In fed mice (fed EL4 groups and Eµ-*Myc* mice) TBR did not increase significantly after 60 min p.i., whereas in fasted mice (fasted EL4 groups and Colo205 mice) TBR continued to increase for at least 170 min p.i. (Fig. 4b). TBR measurements had greater variance than SUV_mean_ with CV ranging from 0.18 – 0.59 for each group, while SUV_mean_ ranged from 0.08 – 0.2 (Table 2).

The image-derived arterial input function area under the curve (IDIF_AUC_) measurements for the 2 min time frames (first 60 min) had a mean value of 2.23 × 10^7^ ± 1.1 × 10^7^ Bq/mL min^−1^ with a CV of 0.50. Using the SUV_mean_ of the blood region of interest (i.e. correcting the measured activity for body weight and injected dose) resulted in a mean IDIF_AUC_ of 39.3 ± 7.8 and a CV of 0.2 (*n* = 26).

In EL4 tumour-bearing mice further increases in tumour uptake were demonstrated between 60 – 170 min in fasted mice but not fed mice regardless of warming status (Fig. 3b). Colo205 tumour-bearing mice also demonstrated this pattern of continued uptake after 60 min, although only the TBR measurements reached statistical significance. In autochthonous Eµ-*Myc* tumours uptake was demonstrated up to 60 min but plateaued thereafter (Fig. 4b).

### Temporal Comparison Between Tumour SUV_mean_ and Influx Constant (K_i_)

Tumour [^18^F]FDG influx rate constants (*K*_*i*_) were calculated from the linear portion of Patlak graphs (between 20—60 min) using an image-derived input function (IDIF) from the inferior vena cava / aorta. Linear regression was used to calculate Patlak *K*_*i*_ (*r*^2^ = 0.92 + 0.11; *n* = 26).

**Fig. 5:**
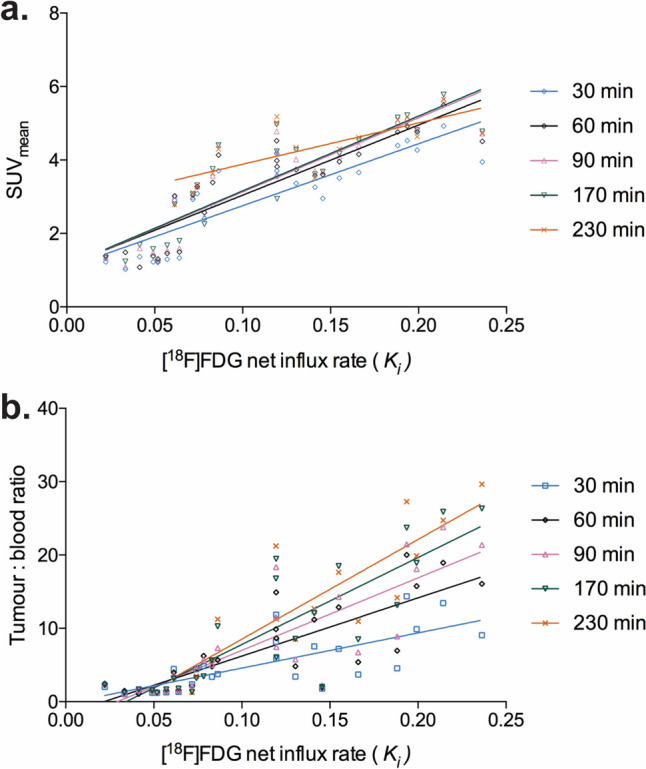
Comparison of tumour *K*_*i*_ and SUV_mean_ measured at different times. RT; room temperature

The ability of static metrics (SUV_mean_ and TBR) to accurately describe the tumour influx constant (*K*_*i*_) were compared over time. There was no significant difference between tumour *K*_*i*_’s predicted from the SUV_mean_ at 30, 60, 90, 170 and 230 min (*r*^2^ = 0.74, 0.77, 0.77, 0.77 and 0.55 at 30, 60, 90, 170 and 230 min, respectively; *P* = 0.11, analysis of covariance – ANCOVA) (Fig. 5a). However, the linear regression gradient of TBR vs. *K*_*i*_ increased over time (*r*^2^ = 0.55, 0.67, 0.70, 0.73, 0.75 at 30, 60, 90, 170 and 230 min; *P* = 0.0002, ANCOVA) consistent with an underestimation of tumour uptake at early time points using TBR (Fig. 5b).

### Derivation of a Population Derived Input Function (PBIDIF) for [^18^F]FDG

**Fig. 6:**
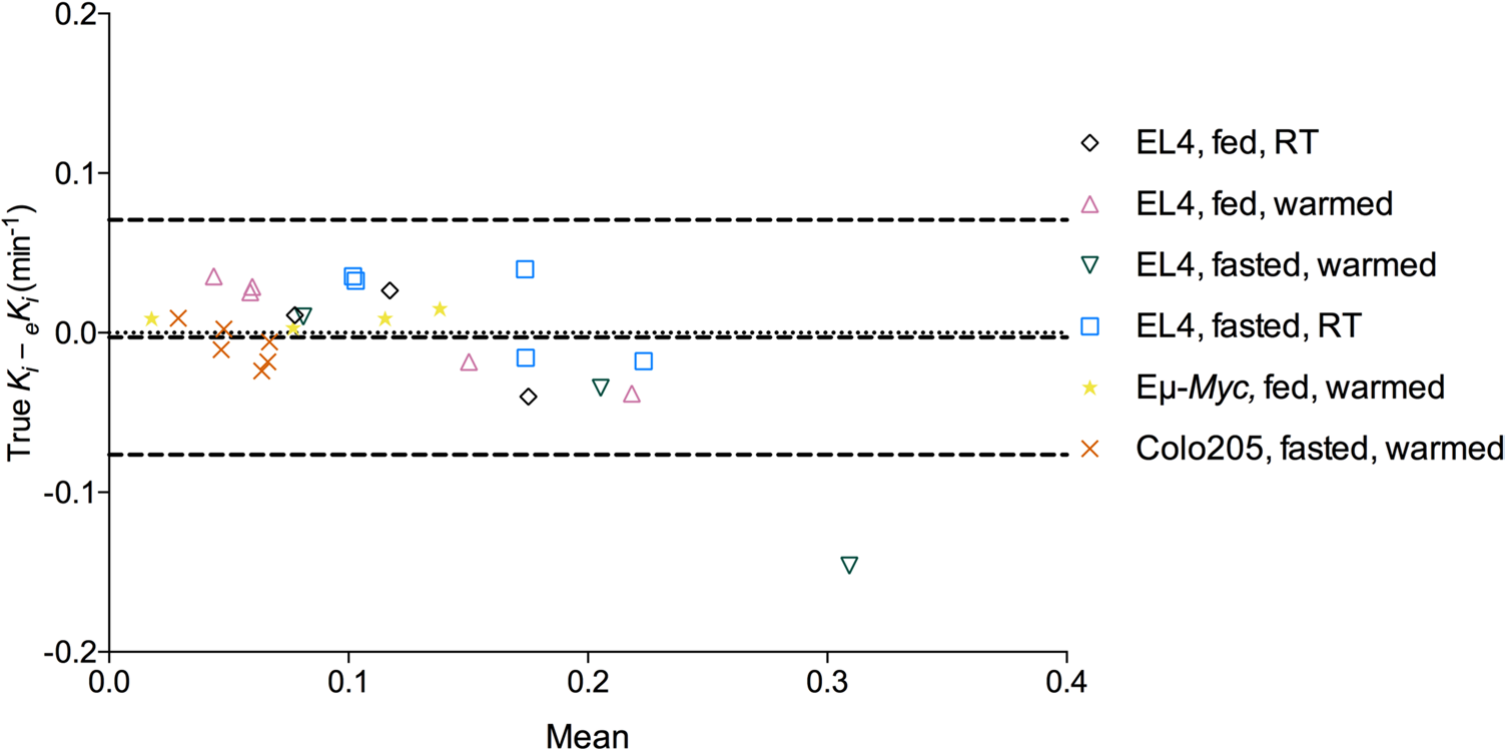
Bland–Altman plot comparing the true *K*_*i*_ and the _e_*K*_*i*_ estimated from the mean PBIDIF. RT; room temperature

The PBIDIF_AUC_ strongly correlated with the IDIF_AUC_ for each mouse during the 20 – 60 min period used for Patlak analysis (*r*^2^ = 0.96 ± 0.02 (*P* < 0.0001, n = 26). An estimated tumour *K*_*i*_ (e*K*_*i*_) calculated using the PBIDIF was compared to the *K*_*i*_ calculated from each individual IDIF. There was good agreement between the *K*_*i*_ and e*K*_*i*_ (*r*^2^ = 0.85, P < 0.0001) with Bland–Altman analysis demonstrating negligible bias (-0.00029) (Fig. 6).

## Discussion

The kinetics of [^18^F]FDG uptake over a more prolonged period has been studied in normal tissues and a number of different diseases in humans with dynamic and multi-time point static acquisitions [9]. Some of the longest delayed acquisitions were performed in glioma patients where tumour-to-background contrast continued to increase up to 8 h post-injection [3]. The potential clinical uses of delayed time-point imaging are improved sensitivity for lesion detection, specificity for malignancy and differentiation of low- and high-grade tumours. Small studies have investigated the use of delayed imaging in multiple organs and cancer types with mixed results. A meta-analysis of using delayed [^18^F]FDG PET for differentiating benign and malignant pulmonary nodules failed to demonstrate a benefit for delayed imaging [10]. Conversely, a meta-analysis of 758 patients concluded that the diagnostic accuracy of [^18^F]FDG PET for mediastinal lymph node metastases was improved by using the retention index of [^18^F]FDG calculated from dual time point imaging [11].

In mouse imaging there have been recent efforts to standardise image acquisition [12, 13]. Tumour uptake typically shows a period of rapid uptake before a plateau. Imaging during the plateau phase would be beneficial for reproducibility and repeatability as small variations in the timing of static imaging will have a minimal effect on the result. However, the majority of pre-clinical studies perform static PET acquisitions no later than 60 min post [^18^F]FDG injection when most tumours, particularly subcutaneous tumours which are poorly perfused, are likely be still in the rapid uptake phase. Furthermore, the dynamic [^18^F]FDG kinetics in mice over a prolonged period have not been defined. For example, in a dual time point study tumour SUV’s increased between 45 and 90 min but, to our knowledge, no previous study has looked at [^18^F]FDG biodistribution beyond this time in small animals [14].

In this study prolonged dynamic [^18^F]FDG-PET acquisitions were performed in three different mouse tumour models, including both subcutaneous and autochthonous models, with the primary aim of defining the kinetics for a range of subcutaneous and autochthonous models under different animal handling conditions. Fasting suppressed myocardial uptake and increased tumour [^18^F]FDG uptake in EL4 tumour-bearing mice, corroborating previous studies [5]. Eµ-*Myc* mice were not fasted as the accompanying clinical features of the model e.g., ascites, pleural effusion, airway compression and cardiac tamponade, rendered them too unwell to undergo fasting and prolonged anaesthesia. The period of fasting prior to mouse studies was aimed to be a minimum of 6 h [7], with variations up to 10 h primarily due to delays in scanner and [^18^F]FDG availability. None of the models demonstrated significant [^18^F]FDG efflux.

Vivariums are typically regulated at approximately 21–25 °C, temperatures that are comfortable for clothed humans. However, this temperature is below the thermoneutral temperature for mice, which is 29–31 °C, and therefore thermogenesis is activated to maintain body temperature and activation of brown fat has been visualised using [^18^F]FDG imaging [5, 15]. Previously it has been demonstrated that warming during the uptake period minimises brown fat [^18^F]FDG uptake. Warming immediately prior to the uptake period has also been proposed for clinical [^18^F]FDG PET imaging. We failed to demonstrate any significant difference in tumour [^18^F]FDG uptake in mice that were warmed or at room temperature prior to injection, and visually there was no significant brown fat uptake in either group. However, warming prior to injection did facilitate tail vein cannulation.

Previous studies of the *repeatability* of [^18^F]FDG-PET in fasted mice reported a 60 min CV in tumours of between 11.4 and 15.4% for the % injected dose per gram (15.1% for SUV_mean_) [16, 17]. In our study the in-group *reproducibility* (CV at 60 min) of tumour SUV_mean_ in fasted mice (EL4, 2 groups; colo205) ranged from 7 – 13%. In the previous studies, test–retest scans were performed 6 h apart. Therefore, the conditioning and Circadian timing differed significantly between scans, which may account for why reproducibility in our study is comparable or better. Using the same methodology repeatability of [^18^F]FLT and [^18^F]FMISO, which are less dependent on animal conditioning, had lower CV’s (4–6%). In EL4 tumour bearing mice that were fed and warmed and Eµ-*Myc* mice the 60 min CV was higher at 20 and 39%, respectively.

Imaging during the initial rapid uptake phase means that relatively small differences in acquisition time will result in large variations in measured uptake. This can be seen in the same patient when multiple acquisitions are performed in different bed positions, a problem that will be alleviated by total body PET [18]. In most tumours and tissues other than brain and liver, the rate of dephosphorylation (*k*_*4*_) of [^18^F]FDG-phosphate is negligible and there is irreversible label trapping. After the initial bolus injection and rapid uptake, *K*_*1*_ and *k*_*2*_ rates start to equilibrate and *k*_*3*_ decreases resulting in a relative plateau in the tissue concentration of [^18^F]FDG. Small animal imaging can be performed in a single bed position and imaging in the plateau period would alleviate the need for precise image timing. In this study, the SUV_mean_ increased in all tumours up to 60 min, with a mean increase from 30 – 60 min between 10.4 ± 4.5%. Beyond 60 min the percentage change in SUV_mean_ reduced to 2.6 ± 3.8% from 60 – 90 min and 1.1 ± 6.7% from 90 – 170 min. These data therefore support performing static tumour imaging in mice at > 60 min post-injection.

In implanted subcutaneous tumours TBR continued to increase up to at least 60 min, an effect that was prolonged in fasted mice, for at least 170 min p.i. in Colo205 and EL4 tumours. In studies where maximising tumour-to-background contrast is paramount then delayed imaging would be appropriate, particularly when imaging tumours that are located within or adjacent to normal tissues that have low rates of uptake and signal is dominated by the blood pool e.g., liver, or significant rates of dephosphorylation [^18^F]FDG-P and washout e.g., brain [3, 19]. The relative insensitivity of SUV values, compared to TBR, to reductions in plasma [^18^F]FDG concentration over time is due to the imaging of unphosphorylated [^18^F]FDG in the tissue compartment [19].

This study used a previously validated method to derive an image derived input function (IDIF) from the inferior vena cava / aorta and generate influx rate constants for tumours (*K*_*i*_*)* [20]. We examined the ROI’s in the left ventricle and carotid / internal jugular veins as alternative sites to define the blood pool, but both of these suffered significantly from partial volume effects and spill over from adjacent structures. A saline flush was not performed following [^18^F]FDG injection to avoid a double peak in the input function. Reconstructing the list-mode data with high temporal resolution to define the peak input function revealed that in time frames < 2 min there was a significant underestimation of activity in a [^18^F]FDG phantom included in the acquisitions. The need to define the peak of the arterial input function was obviated by using Patlak analysis, which is relatively insensitive to low temporal resolution [21].

There was good correlation between tumour SUV_mean_ and *K*_*i*_ indicating that the tumour [^18^F]FDG concentration was dominated by irreversible uptake in the 20–60 min period used for Patlak analysis [22]. Given the increases in SUV_mean_ that occurred over time we hypothesised that SUV_mean_ measured at earlier times would underestimate *K*_*i*_ but this was not the case.

There was good agreement between tumour *K*_*i*_’s calculated from the PBIDIF and individual IDIF’s. This would permit use of a PBIDIF to derive kinetic data where scanning of the whole uptake period cannot, or has not, been performed, or where a ROI cannot be defined over a suitable vessel. To be applied more generally this result will need to be externally validated, particularly if a PBIDIF were to be used with data generated on instruments from other vendors [12].

With prolonged scanning there were three considerations for the injected dose, (a) injecting doses that did not cause initial detector saturation, (b) maintained sufficient count rates for accurate quantification and image quality late in the acquisition when over two half-lives had elapsed and (c) not exceeding recommended injection volumes [13]. The dose injected was similar to that in with previous studies using the NanoScan PET/CT and detector linearity was confirmed using a [^18^F]FDG phantom in the field of view of each study [23].

This study had several limitations. The low numbers of mice in some of the groups limited some of the statistical conclusions that could be made. The lack of blood sampling meant that the study had to use the image derived input function method of Lanz et al. [20]. Kinetic analysis was further restricted to Patlak analysis because short time frames could not be reconstructed, resulting in underestimation of the arterial input function. With the exception of the brain, the ROIs included in the study are defined from the PET imaging and includes all tissues that demonstrated significant irreversible uptake (tumour, myocardium, Harderian gland) and those that demonstrated particularly high perfusion (liver, brain) and excretion (kidneys). The unenhanced CT acquired for the purposes of attenuation correction had poor soft tissue contrast and reliably differentiating organs in the abdomen was challenging, therefore these were not included. Other challenges to defining ROIs included spill-in effects from nearby organs e.g., lungs contaminated by spill-in from the heart and abdominal organs from the urinary tract.

## Conclusions

This study investigated the kinetics of [^18^F]FDG biodistribution over 3 to 4 h post-injection in subcutaneous and autochthonous murine tumour models. Fasting increased tumour [^18^F]FDG uptake but warming mice to their thermoneutral temperature prior to [^18^F]FDG injection did not have a significant effect. Delaying static tumour imaging until at least 60 min post-injection and after the rapid [^18^F]FDG uptake period in all animal handling conditions minimises the errors caused by small variations in imaging time. At imaging times < 60 min, although SUV_mean_ still reflects tumour *K*_*i*_, small variations in post-injection imaging time can cause much larger variations in static measurements of [^18^F]FDG uptake due to the rapid influx at early timepoints. Late delayed imaging (> 90 min post injection) may be of benefit where maximal tumour-to-background contrast is required, particularly in subcutaneous tumours or when imaging tumours in organs that demonstrate minimal or reversible uptake.

## Supplementary Information

Below is the link to the electronic supplementary material.

Supplementary file1 Comparison of liver time activity curves in EL4 tumour-bearing mice under different conditions. RT; room temperature (TIFF 250 KB)

Supplementary file2 Coefficient of variation (CV) values for tumour and myocardium SUVmean over time for each tumour type and EL4 tumour types under different animal handling conditions. RT; room temperature (TIF 1442 KB)

## Data Availability

The data that support the findings of this study are openly available in the University of Cambridge Apollo database 10.17863/CAM.112730.
