# Supplementary figures and images for: Optimisation of Animal Handing and Timing of 2-deoxy-2-[18F]fluoro-D-glucose PET Tumour Imaging in Mice
Source: Mol Imaging Biol. 2024 Nov 11;26(6):965–76. doi: 10.1007/s11307-024-01956-4 (PMC11634969; doi:10.1007/s11307-024-01956-4)

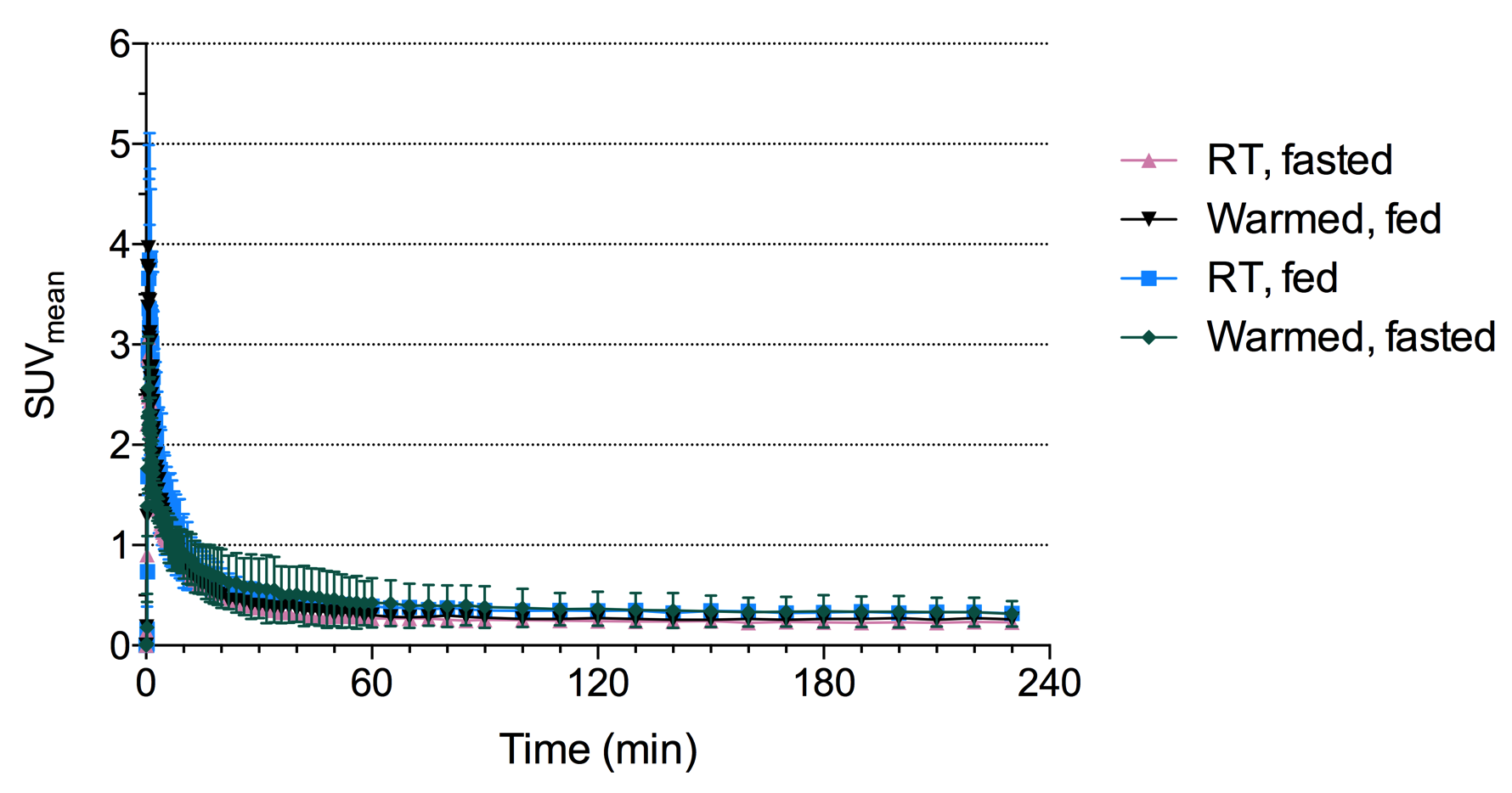

Supplement: Supplementary file 1 — Supplementary file1 Comparison of liver time activity curves in EL4 tumour-bearing mice under different conditions. RT; room temperature (TIFF 250 KB) [file 11307_2024_1956_MOESM1_ESM.tiff]

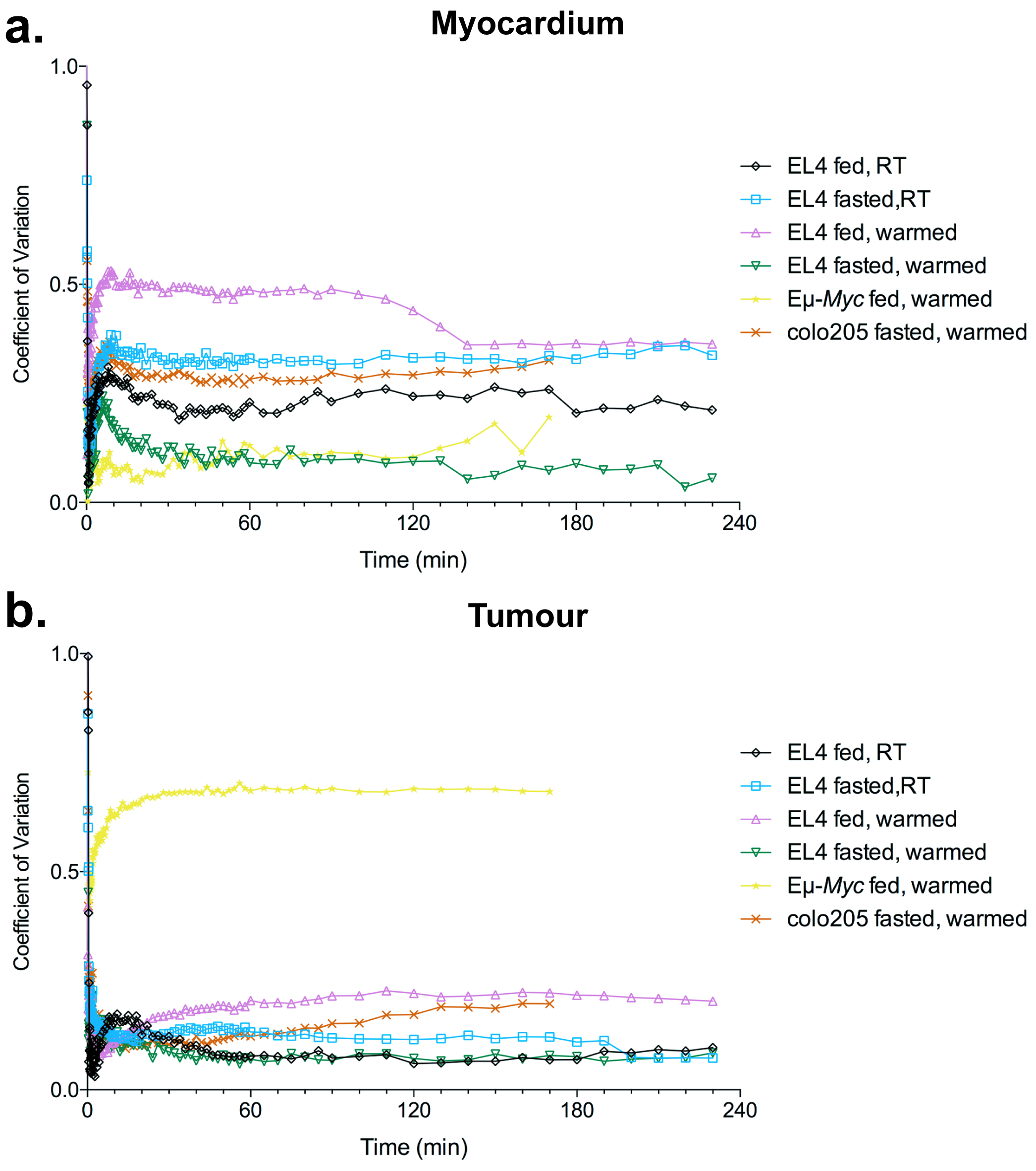

Supplement: Supplementary file 2 — Supplementary file2 Coefficient of variation (CV) values for tumour and myocardium SUVmean over time for each tumour type and EL4 tumour types under different animal handling conditions. RT; room temperature (TIF 1442 KB) [file 11307_2024_1956_MOESM2_ESM.tif]
